# Co-administration of Chrysin and Luteolin with Cisplatin and Topotecan Exhibits a Variable Therapeutic Value in Human Cancer Cells, HeLa

**DOI:** 10.1021/acsomega.3c04443

**Published:** 2023-10-27

**Authors:** Ritu Raina, Arif Hussain, Abdulmajeed G. Almutary, Shafiul Haque, Tasleem Raza, Ashley Cletus D’Souza, Sachin Subramani, Akash Sajeevan

**Affiliations:** † School of Life Sciences, Manipal Academy of Higher of Education, Academic City 345050, Dubai, United Arab Emirates; ‡ Department of Biomedical Sciences, College of Health Sciences, Abu Dhabi University, Khalifa City, Abu Dhabi 51072, United Arab Emirates; § Department of Medical Biotechnology, College of Applied Medical Sciences, Qassim University, Buraydah 52571, Saudi Arabia; ∥ Research and Scientific Studies Unit, College of Nursing and Allied Health Sciences, Jazan University, Jazan 45142, Saudi Arabia; ⊥ Department of Biochemistry, Era’s Lucknow Medical College and Hospital, Lucknow 226003, India

## Abstract

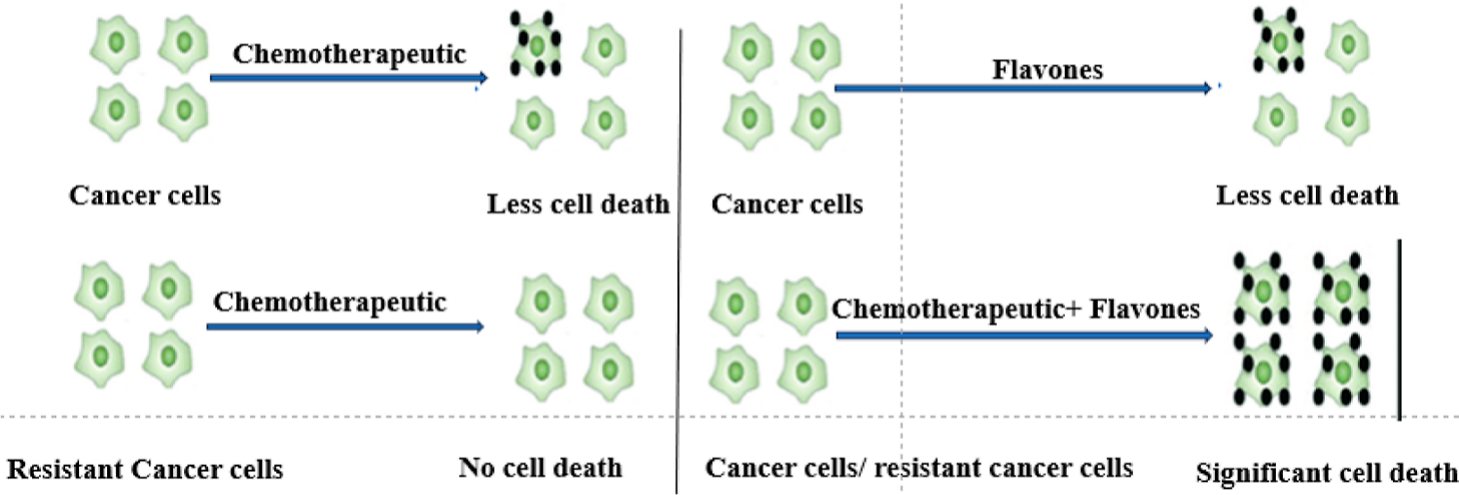

Combinational treatment is a promising strategy for better cancer treatment outcomes. Chrysin and luteolin have demonstrated effective anticancer activity. Cisplatin and topotecan are commonly used for the treatment of human cancers. However, various side effects including drug resistance are an imperative restriction to use them as pharmacological therapy. Therefore, the aim was to use these agents in combination with flavones for better efficacy. In the present study, it was found that the combination of chrysin and cisplatin and luteolin and cisplatin significantly improved the anticancer effect as both the combinations showed synergistic interactions [combinational index (CI < 1)]. Remarkably, the combination of chrysin and luteolin with topotecan depicted the antagonistic interaction (CI > 1). Further, increased expression of the pro-apoptotic proteins Bax and caspase 8 and the inhibition of the antiapoptotic protein Bcl-2 were instituted in the synergistic doses (chrysin + cisplatin and luteolin + cisplatin), hence promoting apoptosis. Also, it was found that the synergistic combination inhibited the migration of HeLa cells by downregulation of metalloproteases and upregulation of TIMPs. However, there are no significant changes depicted in the antagonistic combinations which support their role in their antagonistic effects. Based on these results, it can be inferred that the two or more drug combinations need to be explored well for their interaction to enhance the therapeutic outcomes.

## 1 Introduction

Cisplatin is a chemotherapy drug that is frequently used to treat a variety of human malignancies, including head and neck, testicular, ovarian, cervical, lung, and colorectal cancer.^1,2^ It induces cell death by causing DNA damage and impairing the function of MDM2, leading to apoptosis via the p53 pathway.^3^ Topotecan is an effective drug for the treatment of reproductive cancers.^4^ It has shown results alone and in combination with cisplatin in cervical cancer.^3^ Topotecan induces cell death in cervical cancer by activation of DAPK1.^3^ Topotecan/cisplatin, in spite of being an effective antineoplastic treatment, elicits a lot of side effects.^1,2,5^ Chemotherapy resistance is a key factor that limits the therapeutic effect of these agents, but owing to their potent and extensive therapeutic benefits against various malignancies, development of a new combination strategy which improves the severity of these chemotherapeutics is being studied extensively.^6,7^ Therefore, the combination of chemotherapeutics with flavones might be part of a plausible cancer treatment strategy.

Flavones, a subclass of flavonoids present in various fruits and vegetables, are known to have antiviral, antioxidant, and anticancer activities.^8,9^ Flavones employ their antineoplastic effect via different pathways such as programmed cell death initiation, cell cycle arrest, removal of reactive oxygen species, inhibition of vascular endothelial growth factor, and fibroblast growth factor-mediated angiogenesis.^10−21^ Studies have established that flavones impede tumor growth in cancer cells by triggering the apoptotic path via diverse mechanisms.^10,22,23^ Furthermore, flavones have a high safety profile, with no opposing side effects.^24,25^

Chrysin (5,7-dihydroxyflavone) and luteolin (3,4,5,7-tetrahydroxyflavone) are flavones present in fruits, vegetables, honey, propolis, etc., and are shown to have antioxidative and anti-inflammatory effects.^26−28^ Luteolin and chrysin increase superoxide dismutase, glutathione peroxidase, and catalase levels and thus exhibit antioxidant effects. Furthermore, chrysin inhibits inflammation because it decreases cyclooxygenase-2 levels.^29^ In vitro and in vivo studies of chrysin and luteolin have suggested various mechanisms for their anticancer effects.^14,30−35^ They induce cytotoxicity and persuade apoptosis by various pathways.^20,31,33,34,36,37^ Luteolin and chrysin affects the PI3K/Akt pathway; caspase 3, 7, 9, and 8 activation; the tumor necrosis factor-α pathway; and mitochondrial membrane depolarization.^20,28,33,38,39^ Additionally, chrysin and luteolin inhibit histone deacetylase at the activity and transcript level and modulate the DNA methylation of various TSGs.^40−42^ Remarkably, luteolin and chrysin have also shown inhibition of migration and modulation of epigenetic pathways in different cancer cells.^14,41−45^

Notably, the effect of luteolin and chrysin as a single agent for cancer treatment is further enhanced in combination by sensitizing cancer cells to chemotherapeutics.^1,6,8,46^ Pretreatment with chrysin increased the therapeutic efficiency of DOX to induce apoptosis in HepG2 cell line and animal models.^46^ Similarly, luteolin made the resistant colorectal cells sensitive to oxaliplatin, cisplatin, and doxorubicin.^8^ Luteolin and oxaliplatin synergistically inhibited cell proliferation of HCT116 xenograft tumors by increasing PARP and p53 promoting apoptotic death and reducing the cytoprotective ability of HO-1.^47^ Consistently, luteolin with oxaliplatin together decreased proliferation in gastric cancer cells (SGC-7901) via the cytc/caspase pathway, resulting in the cleavage of caspase 3 and reduced Bcl-2/Bax ratio.^48^ Chrysin has also increased the therapeutic effect of doxorubicin in doxorubicin-resistant HCC cells (BEL-7402/ADM) by modulation of NRf2 and PI3K/AKT/MAPK pathways.^49^ Chrysin significantly heightened the therapeutic outcome of cisplatin treatment in HepG2 cells by p53 modulation.^1^ Thus, the present study was aimed to understand the anticancer effect of chrysin and luteolin in combination with cisplatin and topotecan.

## 2 Materials and Methods

### 2.1 Cell Culture

The human cervical carcinoma (HeLa) cell line obtained from Dr. Tahir A. Rizvi, UAE University, Al-Ain, UAE, was used in this study. It was cultured using Dulbecco’s modified Eagle’s media (DMEM) (PAN Biotech, Germany), which is reconstituted using 10% fetal bovine serum (FBS), amphotericin (Sigma, Merck, KgaA.), glutamine, and 100× pen-strep (Sigma-Aldrich, Merck, KgaA.) incubated at 37 °C with 5% CO_2_ and high humidity.

### 2.2 Drug Preparation

Luteolin and chrysin were obtained from Sigma-Aldrich, Merck, and KgaA. The main stocks of 69.84 and 78.67 mM luteolin and chrysin, respectively, were prepared in DMSO. A 1 mM working concentration using complete media was prepared from the main stocks, and respective dilutions were used for drug treatment. In previous studies from our lab, the IC50 value was found to be 1.25 and 5 μM (data published) for topotecan and cisplatin, respectively.^2,50^ Similarly, based on our previous studies, the IC50 values of chrysin and luteolin are 15 and 20 μM, respectively.^20,33^ The sublethal concentrations were used for these agents based on previous studies at our lab. The concentrations of luteolin used were L1—2.5, L-2—5, and L3—10 μM, and for chrysin, CH1—2, CH2—4, and CH3—8 μM were used. Cisplatin and topotecan were also obtained from Sigma-Aldrich Merck, KgaA. From the main stock of 1.67 mM cisplatin, 100 μM working solution was prepared in complete media; using this, respective dilutions such as C1—0.5, C2—1.5, and C3—3.0 μM were used for combination studies. Similarly, from the main stock of 2.2 mM topotecan, 10 μM substock was made in complete media, and the respective concentrations of topotecan prepared were T1—0.025, T2—0.05, and T3—0.1 μM as given in Table 1.

**Table 1:** Combinations of Chrysin and Luteolin with Cisplatin and Topotecan Are Enumerated together with Their Short Form

			chrysin			luteolin		
			CH1	CH2	CH3	L1	L2	L3
concentrations of agents used individually			2 μM	4 μM	8 μM	2.5 μM	5 μM	10 μM
cisplatin	C1	0.5 μM	C1CH1	C1CH2	C1CH3	C1L1	C1L2	C1L3
	C2	1.5 μM	C2CH1	C2CH2	C2CH3	C2L1	C2L2	C2L3
	C3	3 μM	C3CH1	C3CH 2	C3CH 3	C3L1	C3L2	C3L3
topotecan	T1	0.025 μM	T1CH1	T1CH2	T1CH 3	T1L1	T1L2	T1L3
	T2	0.05 μM	T2CH1	T2CH2	T2CH3	T2L1	T2L2	T2L3
	T3	0.1 μM	T3CH1	T3CH2	T3CH3	T3L1	T3L2	T3L3

### 2.3 Viability Assay

The cell viability was measured by using the 3-(4,5-dimethyl-2-thiazolyl)-2,5-diphenyl-2-*H*-tetrazolium bromide (MTT) assay. HeLa cells (8 × 10^3^ per well) were seeded into 96-well plates. After achieving an 80% growth confluency, the respective drugs and their combinations were administered into the wells. The concentrations of cisplatin used were 0.5, 1.5, and 3 μM, while the concentrations of luteolin used were 2.5, 5, and 10 μM and that of chrysin were 2, 4, and 8 μM. The concentrations of topotecan used were 0.025, 0.05, and 0.1 μM. Table 1 shows the respective individual and combination treatments. After 48 h of treatment, 10 μL of MTT (a final concentration of 0.5 mg/mL) + 200 μL of DMEM without FBS was added to each well and incubated for 2 h. The media were then decanted, and the wells were then replenished with 100 μL of DMSO, followed by a 20 min incubation with shaking. The absorbance of the plate was measured by using an ELISA reader (BioTek, USA) at a wavelength of 570 nm. The experiment was performed in triplicate, and the average is represented in the data. The % cell viability was calculated using the following formula
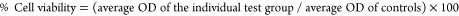


### 2.4 Calculation of Combination Effects of Cisplatin and Topotecan with Chrysin and Luteolin

The combination index (CI) was calculated to indicate the effect of the drugs in combination and to determine the drug interaction as described in our previous combination study.^2^ The formula used to calculate CI is as follows
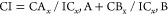
where CA_*x*_ and CB_*x*_ refer to the concentrations of drugs A and B in combination to achieve the *X* % drug effect, respectively. IC_*x*_, A and IC_*x*_, B refer to individual drug concentrations that cause the identical effect. A CI value <1, = 1, or >1 indicates synergy, additivity, or antagonism of the two drugs, respectively.

### 2.5 Mitochondrial Potential Assay

The tetramethylrhodamine (TMRE, ethyl ester) mitochondrial membrane potential assay kit (ab113852) was obtained from ABCAM (Abcam, Cambridge, UK). The kit is used for observing the variations in the mitochondrial membrane potential in live cells using spectrophotometric analysis (Synergy H1 Bioteck Plate Reader, USA) and fluorescence microscopy (Olympus, USA). Nearly 5 × 10^3^ cells were plated in a 96-clear bottom plate and treated with cisplatin (C2), chrysin (CH2), luteolin (L2), and topotecan (T2) individually and in combination with C2L2 (1.5, 5 μM), C2CH2 (1.5, 4 μM), T2CH2 (0.05, 4 μM), and T2L2 (0.05, 5 μM) for 48 h. Following the treatment, TMRE was added to the control cells, treated cells, and negative control cells (cells treated with FCCP for 30 min) and incubated for 20 min at 37 °C. Afterward, the measurement of fluorescence was taken using microplate spectrophotometry (*E*_x_/*E*_m_ = 549/575 nm). Pictures were taken at 40× magnification using a Progress Fluorescent Microscope (Olympus, USA). The experiments were repeated thrice, and % fluorescence was depicted as a graph showing mean ± SD (*p* ≤ 0.05). The percentage of fluorescence in treated samples in comparison to the control samples was calculated.

### 2.6 Scratch Wound Assay

The migration of cancer cells under the effect of cisplatin, topotecan, luteolin, and chrysin individually and in combination treatment was examined by the scratch wound assay. About 1.8 × 10^5^ cells per well were seeded into a 12-well plate and cultured until wells were confluent. A 10 μL pipette tip was used to create a vertical cell-free line through the well. Individual wells were treated with the following drug combinations: C2, T2, CH2, L2, T2L2, C2L2, C2CH2, and T2CH2. The untreated cell group was considered a control. The migration of the cells was monitored and imaged using an inverted microscope at 0 and 48 h. The wound width was measured, and the % wound closure/widening was calculated and graphically represented.

### 2.7 Expression Analysis of Apoptosis and Migration-Related Genes Using Quantitative PCR

The apoptotic and antimigratory behavior of the cotreated combinations was confirmed at the molecular level through analysis of the expression of various genes. After performing a 48 h drug treatment with selected combinations, the total RNA was isolated using a GenElute Mammalian Genomic Total RNA extraction kit (Sigma, USA) from the untreated cells and cells treated with concentrations C2, L2, CH2 and C2L2, C2CH2, and cDNA synthesis was done using 2 μg of RNA and the measurement of components and temperature as per the protocol FIRE Script RT cDNA Synthesis KIT.

Two housekeeping genes were used as reference genes, which are GAPDH and β-actin (ACTB). The obtained gene expression profiles may differ depending on the selection of the housekeeping gene as a reference gene.^51^ The primers used in this study are TIMP1, TIMP2, MMP2, MMP9, Bcl-2, Bax, CASP3, CASP8, CASP9, GAPDH, and ACTB. The lyophilized powders were dissolved in appropriate volumes of nuclease-free water to obtain a molarity of 100 μM. For the conventional PCR reactions, 10 μM primer dilutions are used. 100 μL of 10 μM primer dilutions is made by adding 10 μL of 100 μM primer suspension and 90 μL of nuclease-free water. For RT-qPCRs, 5 μM primer dilutions are used. The master mix used for this experiment is 5X HOT FIREPol EvaGreen qPCR SuperMix (Solis BioDyne). cDNA samples of individual drugs and combinations are taken as per Table 3. The gene-specific primers used are from Table 2.

**Table 2:** Gene-Specific Primers Used for qPCR Analysis of Genes Related to Apoptosis and Migration

	primer sequences		size
primers	forward primer	reverse primer	bp
**TIMP1**	**5**′GACGGCCTTCTGCAATTCC3′	**5**′GTATAAGGTGGTCTGGTTGACTTCTG3′	**79**
**TIMP2**	**5′GAGCCTGAACCACAGGTACCA3′**	**5**′AGGAGATGTAGCACGGGATCA3′	**77**
**MMP2**	5′GGCCCTGTCACTCCTGAGAT3′	**5′GGCATCCAGGTTATCGGGGA3′**	**474**
**MMP9**	**5′CCTGCCAGTTTCCATTCATC3′**	**5′GCCATTCACGTCGTCCTTAT3′**	**455**
**Bcl-2**	**5′ATCGCCCTGTGGATGACTGAG3′**	5′CAGCCAGGAGAAATCAAACAGAGG3′	**129**
**Bax**	5′GGACGAACTGGACAGTAACATGG3′	5′GCAAAGTAGAAAAGGGCGACAAC3′	**150**
**CASP3**	5′CCTGGTTATTATTCTTGGCGAAA3′	5′GCACAAAGCGACTGGATGAA3′	**62**
**CASP8**	5′CAGGCAGGGCTCAAATTTCT3′	5′TCTGCTCACTTCTTCTGAAATCTGA3′	**65**
**CASP9**	5′TGCTGAGCAGCGAGCTGTT3′	5′AGCCTGCCCGCTGGAT3′	**57**
**GAPDH**	**5**′TGTTCGTCATGGGTGTGAAC3′	5′ATGGCATGGACTGTGGTCAT3′	**154**
**ACTB**	5′TCTGGCACCACACCTTCTACAATG3′	5′AGCACAGCCTGGATAGCAACG3′	**166**

The parameters set are (i) denaturation at 95 °C for 15 s, (ii) annealing at 52–54 °C for 20 s, and (iii) elongation at 72 °C for 20 s. The PCR array was run on QuantStudio3 and analyzed by the ΔΔC_T_ method by using DataAssist software from Thermo Fisher. The RQ/FC value indicated the fold change in gene expression against the untreated control.

#### 2.7.1 Statistical Analysis

SPSS software (version 21.0) was used to perform statistical evaluation. The data was investigated using one-way ANOVA, and further verification by Tukey’s HSD post hoc analysis was done. All experiments were done three times, and values were taken as the mean of three experiments ± SD (*P* ≤ 0.05).

## 3 Results

### 3.1 Chrysin and Luteolin Enhance the Action of Cisplatin while Diminish the Topotecan Effect against HeLa Cells

**Figure 1:**
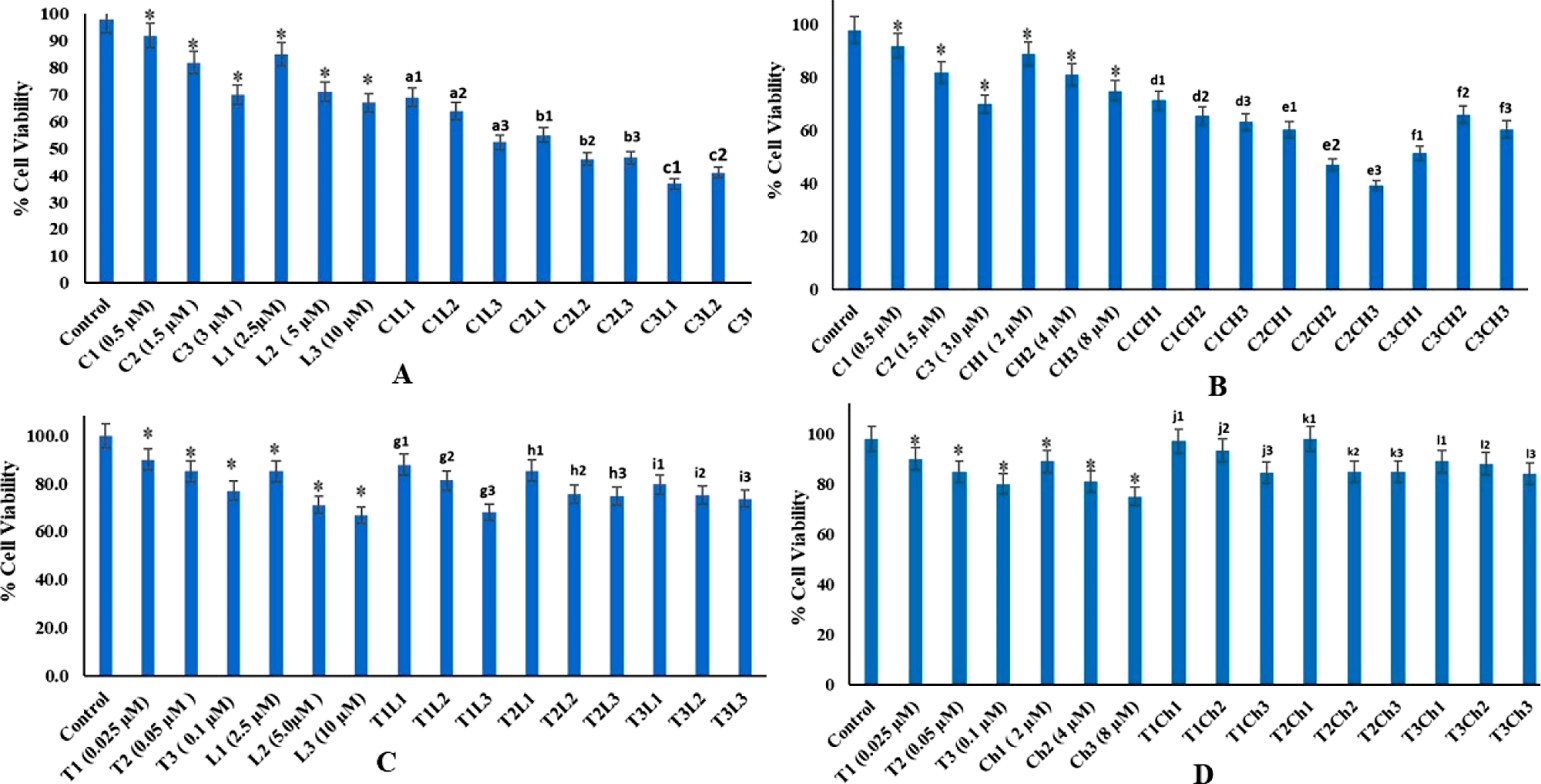
(A) Viability of Hela cells after treatment with different concentrations of cisplatin and luteolin individually and in combination. (B) Viability of Hela cells after treatment with different concentrations of cisplatin and chrysin individually and in combination (C) Viability of Hela cells after treatment with different concentrations of topotecan and luteolin individually and in combination. (D) Viability of Hela cells after treatment with different concentrations of topotecan and chrysin individually and in combination. All results were taken as mean ± SD, *p* < 0.05, and are indicative of three independent studies. For designation of statistical significance in individual treatments, an asterisk is used, whereas for combination treatments, alphabets are used. For C1 and different concentrations of luteolin, a1, a2, and a3 are used; for C2 and different concentrations of luteolin, b1, b2, and b3 are used; and for C3 and LI, L2, and L3, c1, c2, and c3 are used. For C1 and different concentrations of chrysin, d1, d2, and d3 are used; for C2 and concentrations of chrysin, e1, e2, and e3 are used; and for C3 and chrysin combinations, f1, f2, and f3 are used. For T1 and L1, L2, and L3—g1, g2, and g3; for T2 and L1, L2, and L3—h1, h2, and h3; and for T3 and L1, L2, and L3—i1, i2, and i3. For T1 and CH1, CH2, and CH3—j1, j2, and j3; for T2 and CH1, CH2, and CH3—k1, k2, and k3; and for T3 and CH1, CH2, and CH3—l1, l2, and l3.

The cell viability following treatment with chrysin CH1 (2 μM), CH2 (4 μM), and CH3 (8 μM) was 89, 81, and 75%, and C1 demonstrated 92% viability, while in combination with increasing concentration of chrysin, the viability dropped to 71.0, 65.6, and 62.2% for C1CHI, C1CH2, and C1CH3, respectively (Table 3). However, after the treatment with luteolin L1 (2.5 μM), L2 (5 μM), and L3 (10 μM), the cell viability was 85, 71, and 67%, respectively. Similarly, for a combination of cisplatin (C1) with luteolin, the cell survival dropped to 69, 64, and 52.3% for C1LI, C1L2, and C1L3, respectively. After simultaneous exposure of HeLa cells to sublethal doses of cisplatin and chrysin; cisplatin and luteolin brought about a synergistic decrease in cell survival. Cell viability was seen to be much lower than the viability of HeLa cells when treated with these agents individually. The CI was calculated for each of the combinations and found to be less than 1 (Figure 1A,B) except that the highest dose of cisplatin (3 μM) with CH2 and CH3 of chrysin showed antagonism. The lowest viability and CI index was for C2CH2 and C2L2.

**Table 3:** Individual Drug Concentrations and Combinations along with Their % Viability and CI

			chrysin			luteolin		
			CH1	CH2	CH3	L1	L2	L3
concentrations of agents used individually			89%	81%	75%	85.0%	71.0%	67.0%
cisplatin	C1	92.0%	C1CH1	C1CH2	C1CH3	C1L1	C1L2	C1L3
			71%	65.6%	62.2%	69.0%	64.0%	52.3%
			0.61	0.67	0.84	0.66	0.65	0.59
	C2	82.0%	C2CH1	C2CH2	C2CH3	C2L1	C2L2	C2L3
			60.4%	46.7%	39.2%	55.0%	46.0%	46.7%
			0.76	0.43	0.52	0.48	0.43	0.63
	C3	70.0%	C3CH1	C3CH2	C3CH3	C3L1	C3L2	C3L3
			51.4%	66%	60.5%	36.8%	41.0%	42.0%
			0.68	2.3	1.84	0.44	0.54	0.68
topotecan	T1	90.0%	T1CH1	T1CH2	T1CH3	T1L1	T1L2	T1L3
			97%	93%	84%	87.9%	81.0%	68.3%
			4.0	5.0	4.8	3.16	2.25	1.43
	T2	85.0%	T2CH1	T2CH2	T2CH3	T2L1	T2L2	T2L3
			98%	85%	85%	85.4%	75.5%	74.9%
			6.6	2.05	3.13	2.42	1.6	2.9
	T3	80.0%	T3CH1	T3CH2	T3CH3	T3L1	T3L2	T3L3
			89%	88%	84%	79.6%	75.3%	73.7%
			4.8	5.6	4.1	2	2.34	3.37

Interestingly, combined treatment of chrysin and topotecan/luteolin and topotecan mostly increased the cell viability in almost all combinations as the cell viability was found to be higher than the average viability of the two agents used alone, and the CI value was more than 1. Luteolin L3 (10 μM) individually led to 67% viability, while three concentrations of topotecan (T1, T2, and T3) led to a viability of 90, 85, and 80%, respectively. In contrast, combinations of topotecan T1, T2, and T3 with L3 resulted in cell viability more than L3 that is 68.3, 74.9, and 73.8% viability for T1L3, T2L3, and T3L3, respectively, and CI of 1.83, 2.9, and 3.37, respectively, thereby showing antagonism (Table 3). The viability for CH1 was 89%; the combination of T1, T2, and T3 with CH1 depicted 97, 98, and 89% viability, respectively; and the CI much greater than 1 is indicative of antagonism (Figure 1C,D). Similarly, the viability for chrysin (CH3) was 75%, and topotecan (T1, T2, and T3) led to a viability of 90, 85, and 80%, respectively. However, the viability of T1CH3, T2CH3, and T3CH3 was 84, 85, and 84%, respectively, which was much higher than that of CH3, thus indicating that chrysin and topotecan behaved antagonistically (Table 3). The data are quantified as mean ± SD, *p* < 0.05, and are expressed as the average of three independent experiments.

### 3.2 Synergistic Combinations Altered Mitochondrial Potential Significantly as Compared to Individual Treatments

**Figure 2:**
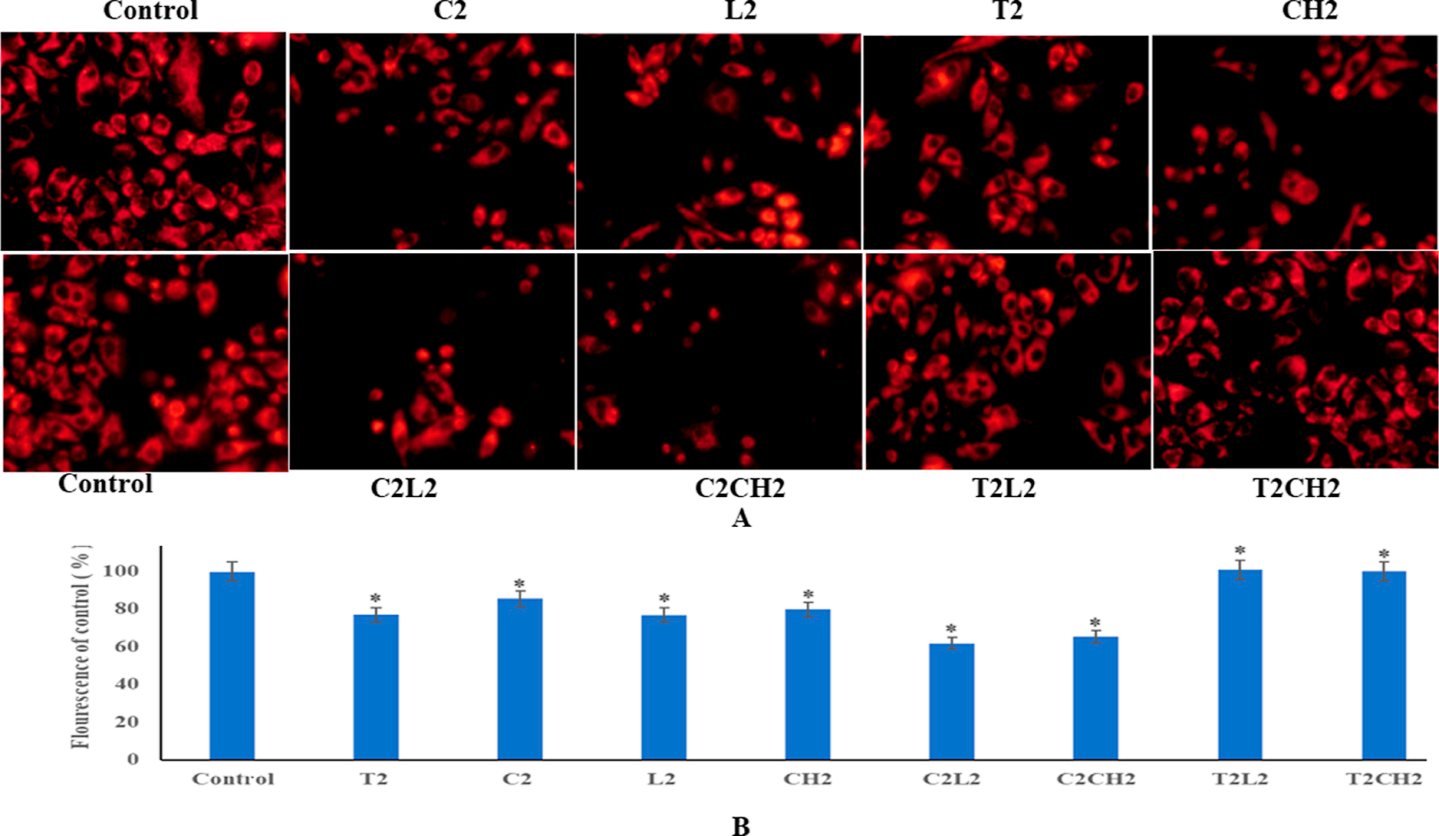
(A) Images depicting the decrease in mitochondrial potential by the combination of cisplatin and luteolin and cisplatin and chrysin and no effect with the combination of luteolin and topotecan and chrysin and topotecan. (B) Graphical representation of decrease in fluorescence compared to the control. The data are quantified as mean ± SD (*p* < 0.05) and are indicative of three independent studies.

The drug combinations of cisplatin (C2) with luteolin L2 (C2L2) and the combination of cisplatin (C2) and chrysin (CH2) C2CH2 exerted a synergistic effect and depicted a decrease in the fluorescence of these combination wells. This demonstrated a significant decrease in mitochondrial potential in combinations in comparison to individual treatments. The drug combinations of topotecan with luteolin and topotecan with chrysin exerted an antagonistic effect. The fluorescence of wells treated with the combinations of luteolin and topotecan and chrysin and topotecan was similar to that of the control wells (Figure 2A,B). The data are specified as mean ± SD, *p* < 0.05, and are indicative of three independent studies.

### 3.3 Luteolin and Chrysin in Combination with Cisplatin Demonstrated Antimigratory Behavior

**Figure 3:**
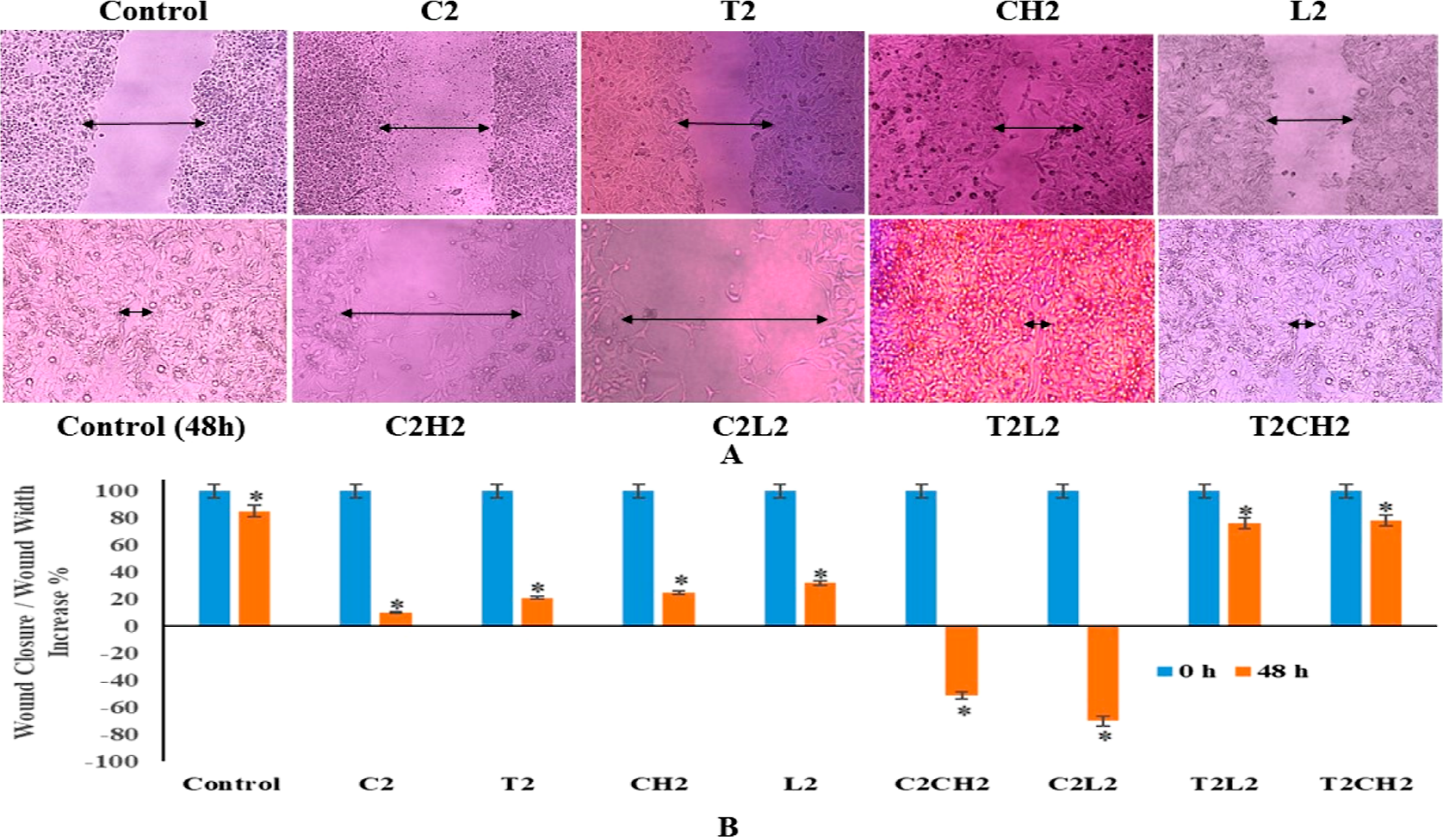
(A) Images show the wound widening with the combination of luteolin and cisplatin and chrysin and cisplatin in comparison to the wound closure in untreated wells and wound closure for luteolin and topotecan and chrysin and topotecan combinations. (B) Graphical representation of wound width. The data are stated as mean ± SD, *p* < 0.05, and are indicative of three independent studies.

The antimigratory effect of HeLa cells treated with individual treatments of C2, L2, and CH2 and the combination of C2L2 and C2CH2 was determined. There was profound synergetic antimigratory effect in C2L2 and C2CH2 which showed 170 and 155% increase in wound width in comparison to individual treatments of C2, L2, and CH2 that depicted 10, 25, and 30% wound closure, respectively. In the untreated controls, there was 80% closure of the wound after 48 h and complete closure after 72 h, whereas the combinations showed a noticeable antimigratory effect on HeLa cells with the cell-free zone remaining unclosed and further widening due to profound cell death (Figure 3A,B).

### 3.4 Luteolin and Chrysin in Combination with Topotecan Showed Antagonistic Effect toward Migration

The combination of T2L2 and T2CH2 showed antagonism, as there was 80% closure of the wound after these combination treatments like the closure of wound in untreated controls after 48 h. The individual treatments of T2, L2, CH2, and C2 did show wound closure of 20, 30, 25, and 10%, respectively, whereas the combinations of T2L2 and T2CH2 behaved as no drug control well (80% closure) (Figure 3A,B).

### 3.5 Synergistic Combinations Demonstrated Enhanced Apoptosis Induction and Antimigratory Behavior in Comparison to the Individual Treatment

**Figure 4:**
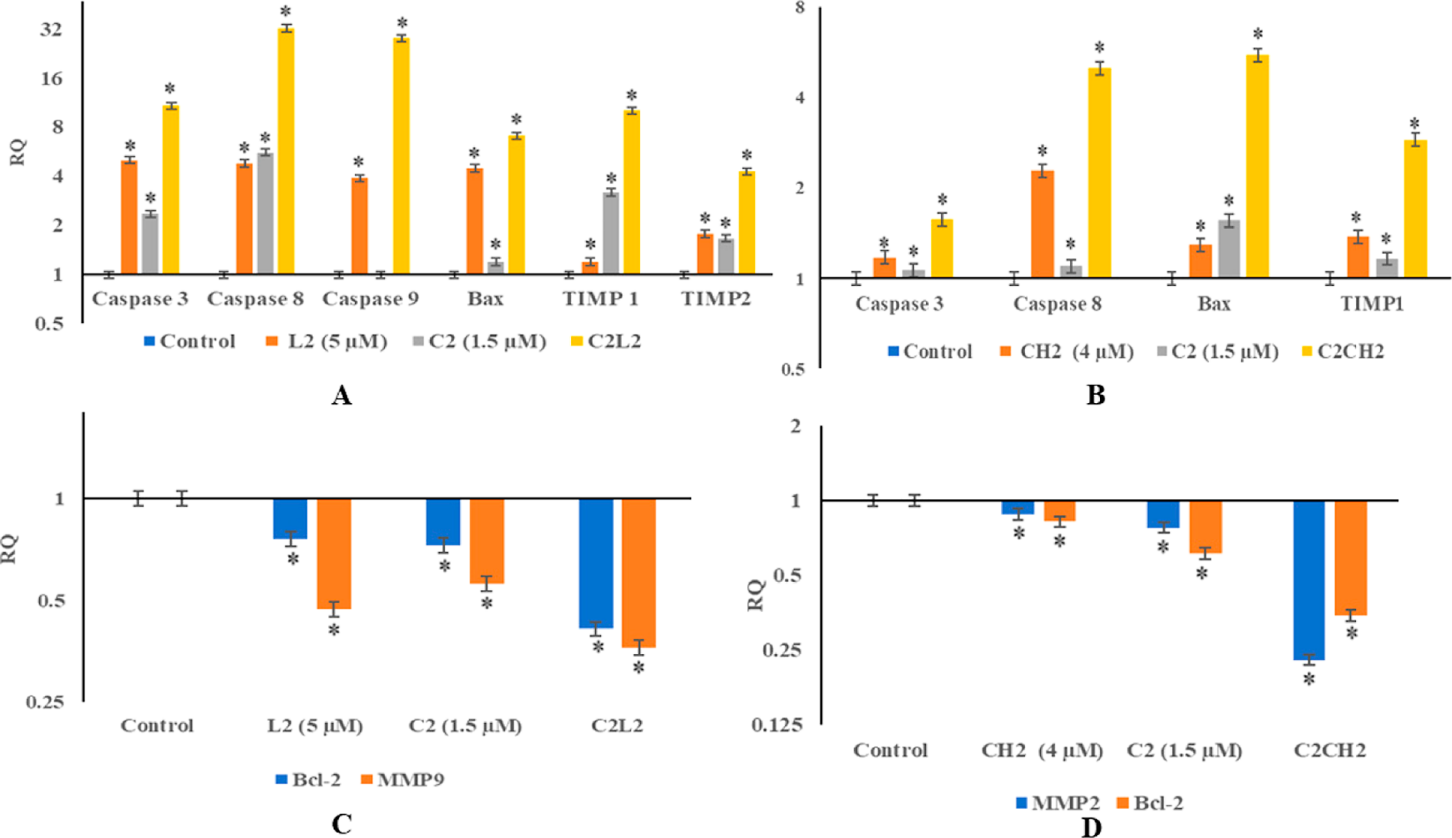
Modulation of apoptotic and migration-related genes. (A) Upregulation of Bax, caspases, and TIMPs by a combination of cisplatin and luteolin. (B) Upregulation of Bax, caspases, and TIMPs by a combination of cisplatin and chrysin. (C) Downregulation of Bcl-2 and MMP9 by a combination of luteolin and cisplatin. (D) Downregulation of Bcl-2 and MMP2 by a combination of chrysin and cisplatin. The data are calculated as mean ± SD, *p* < 0.05, of three independent studies.

Expression analysis was considered only for synergistic treatments as the antagonistic combination did not show any alteration in viability, scratch wound, and mitochondrial potential assays. Hence, these combinations were not pursued further for expression studies to check the effect on apoptosis, migration, and invasion in HeLa cells. Cell treatment with C2CH2 and C2L2 in comparison with individual treatments of C2, L2, and CH2 was performed. Expressions of various genes involved in these hallmarks were studied using a nonsequence-specific DNA dye Eva Green RT-qPCR method. The results indicated that for the synergistic combination of C2L2, the expression of caspases 3 (RQ = 10), caspase 8 (RQ = 28), caspase 9 (RQ = 32), and Bax increased (RQ = 7) many folds in comparison to individual agent treatments of C2 and L2 (Figure 4A). Similarly, C2CH2 upregulated the expression of apoptosis-inducing caspases, i.e., CASP 8 (RQ = 5) CASP 3 (RQ = 1.5), and Bax (RQ = 5.5), in comparison to individual treatments of C2 and CH2 which depicted little upregulation; only in caspase 8, CH2 showed a 2-fold increase (Figure 4B). Similarly, it was also observed that the expression of Bcl-2, an antiapoptotic protein, is downregulated significantly to RQ values of 0.4 and 0.22 by combinations of C2L2 and C2CH2 in comparison to individual treatments of C2, CH2, and L2, which were in the range of 0.7–0.8 (Figure 4C,D).

The observed results also demonstrate that the synergistic combinations of C2CH2 and C2L2 modulate the genes involved in migration and invasion. The expressions of TIMP1 were upregulated by C2H2 (RQ = 3) in comparison to individual treatments of C2 and CH2 (Figure 4A). C2L2 upregulated TIMP1(RQ = 10) and TIMP2 (RQ = 4) as compared to individual treatments of C2 and L2 wherein it depicted RQ between 1.5 and 3 (Figure 4B). The expression of MMP9 was downregulated expressively by C2L2 (RQ = 0.36) (Figure 4C) in comparison to individual treatments of C2 (RQ = 0.56) and L2 (0.46), and the expression of MMP2 was downregulated significantly by C2CH2 (RQ = 0.22) combination, in comparison to the individual treatments of C2 (RQ = 0.77) and CH2 (RQ = 0.88) (Figure 4D).

## 4 Discussion

Cisplatin and topotecan are the utmost effective therapeutic agents extensively used for the treatment of many reproductive cancers including cervical cancer.^6,52−54^ However, drug resistance is a major problem that limits their clinical application. Therefore, combination treatment with new plant-derived agents is an effective approach to overcome drug resistance.^6,55^ Gamut reports have shown that chrysin and luteolin exhibit chemosensitizing properties against various human cancers.^6,49,56^ In this study, we provide experimental evidence that chrysin and luteolin are able to enhance the therapeutic potential of cisplatin in cervical cancer cells (HeLa).

In the current study, first, we evaluated the effect of chosen low concentrations of chrysin, luteolin, cisplatin, and topotecan on HeLa cells. Then, the combinations of cisplatin and luteolin and cisplatin and chrysin were tried. It can be seen that individual tested concentrations of cisplatin, topotecan, chrysin, and luteolin decreased cell proliferation in a concentration-dependent manner (Figure 1A,B) as has been reported by our lab and other laboratories.^2,20,33^ All combinations of cisplatin and chrysin and cisplatin and luteolin showed a synergistic effect (Figure 1A,B). However, the highest combination of chrysin with cisplatin, that is, C3CH2 and C3CH3, depicted antagonism CI > 1 (Table 3). These results suggested that chrysin and luteolin could exert synergistic antiproliferation effect with cisplatin in HeLa cells. Results on the same lines have been observed with luteolin (50 mg/kg body weight) (BW)/day) and oxaliplatin (10 mg/kg BW/day) three times per week for 3 weeks in HCT116 xenografts in Balb mice.^47^ Similar results have been observed with chrysin at 40 μM and cisplatin 5 μg/mL in HepG2 gastric cells and luteolin (10–100 μM) with cisplatin (2 μg/mL).^1,6^ From the tested concentrations, the combination of C2CH2 and C2L2 and T2CH2 and T2L2 was chosen for further assays to confirm what was seen at the viability assay.

Cisplatin is one of the best drugs for the treatment of reproductive cancers, and the mechanisms involved include apoptosis induction. When the cells acquire resistance, the apoptotic pathway is blocked, which reduces the antitumor effect of cisplatin.^1,6,57^ Chrysin and luteolin have been designated to induce apoptosis by inducing nuclear morphological changes, DNA laddering pattern, and modulation of apoptotic genes in a concentration-dependent manner in cancer cells such as human cervical cancer cells, gastric cancer cells, and hepatocellular cancer.^20,33,58−60^ In the present study also, we found that low doses of chrysin and luteolin induced apoptosis alone, but in combination with cisplatin, their effect was more pronounced. This was further proved by the TMRE assay. The results of mitochondrial potential revealed that individual treatments did decrease the membrane potential, but substantial effect was observed in combinations of C2CH2 and C2L2. The fluorescence of C2 was 85% and that of L2 and CH2 was 81 and 80%, respectively, and combinations of C2L2 decreased the fluorescence to 61% and C2CH2 decreased the fluorescence to 65%, thus indicative of the synergistic effect of combinations of cisplatin with chrysin and luteolin (Figure 2A,B). Results on the same lines were observed by other laboratories wherein caspase-dependent apoptosis was significantly augmented by chrysin (40 μM) and cisplatin (5 μg/mL) together in Hep2 cells and luteolin (40 μM) and oxaliplatin (30 μM) in gastric adenocarcinoma cell line (SGC-7901) cells in comparison to individual agents.^6,48^

The migration assay was performed using individual concentrations of C2, L2, CH2, and T2 and a combination of C2L2, C2CH2, T2L2, and T2CH2. C2, L2, and CH2 showed 10, 25, and 32% closure, respectively, whereas C2L2 and C2CH2 showed 150 and 170% widening of the wounds, respectively, thus revealing a marked inhibition in the migratory ability of the cells treated with a combination of C2L2 and C2CH2 with the cell-free zone widening due to cell death as compared to the individual treatments of C2, L2, and CH2 (Figure 3A,B). The control wells showed 80% closure at 48h and complete closure at 72 h. The combinations of T2CH2 and T2L2 showed wound closure similar to control wells almost 80% closure in 48 h (Figure 3A,B), thus indicating the antagonistic interaction of topotecan and chrysin and topotecan and luteolin. The results on the same lines were shown by cisplatin (2 μg/mL) and luteolin (50,100 μM) combination in ovarian cancer cells.^6^

Further, to verify the apoptotic induction by individual agents and a combination of C2L2 and C2CH2, qPCR was performed for apoptotic genes like Bax, Bcl-2, and caspases 3, 8, and 9. Modulation of caspases is a key regulator of self-programmed death.^59^ Bcl-2 and Bax are the most important apoptosis regulating proteins; they act as a rheostat for regulation of apoptosis.^61^ Overexpression of Bcl-2 can inhibit cell apoptosis, lead to resistance to cisplatin, and result in poor prognosis for cancer patients. A recent study has demonstrated that Bcl-2 is overexpressed in cervical cancer.^62^ In our previous study, it can be seen that chrysin and luteolin modulated caspases, Bax and Bcl-2, in HeLa cells.^20,33^ In the present study, it can be seen that individually chrysin and cisplatin upregulated caspases 3, 8, and Bax and downregulated Bcl-2. Remarkably, there was significant modulation when HeLa cells were treated with a combination of C2CH2. Bcl2 reduced to 36% of the control (Figure 4D). Similar results of reduction of Bcl-2 to 35% of the control was observed for C2 L2 (Figure 4C). Caspase 9 did not show upregulation by individual low-dose treatments of C2 and L2 but C2L2 combined treatment increased the caspase 9 by 28 folds. Caspase 3 and caspase 8 increased 10- and 32-fold, respectively, after a combined treatment of C2 L2 (Figure 4A). Individual chrysin and cisplatin did not show much modulation of caspase 3 and caspase 8; however, C2H2-combined treatment depicted 1.5- and 5-fold increase, respectively (Figure 4B). The same results were reported for the combination of luteolin (50 and 100 μM) and cisplatin (2 μg/mL), wherein demonstrating synergistic modulation of Bax and Bcl-2 in ovarian cancer.^6^ Programmed cell death-related genes p53 and p27 were upregulated significantly by a combination of chrysin (25 μM) and docetaxel (2.5 ng/mL) in comparison to individual treatment in NSCLC.^46^

Further, expression of MMP2, MMP9, TIMP1, and TIMP2 was also determined to confirm the findings of the scratch wound assay. In our previous reports, it can be seen that chrysin and luteolin have demonstrated dose-dependent modulation of MMP2 and TIMPs in addition to other migratory pathways and genes.^41,42^ Again, as compared to individual low-dose treatments of C2, L2, and CH2, the combination treatments of C2L2 and C2CH2 depicted a significant increase of antimigratory genes. TIMP1 and TIMP2 were upregulated by 10- and 4-fold after C2L2-combined treatment (Figure 4A) and TIMP1 by 3-fold after C2H2 treatment (Figure 4B). These combinations also showed significant modulation of the migratory gene, C2L2 downregulated MMP9, Bcl-2 to RQ of 0.36 and 0.4, respectively, and C2H2 reduced MMP2 to RQ of 0.22 and Bcl-2 to 0.34 (Figure 4C,D). The combinations of C3L1 and C2CH3 were not considered for molecular studies as the aim of this study was to observe the synergistic combination at lowest doses.

## 5 Conclusions

In this study, chrysin and luteolin in combination with cisplatin displayed enhanced antineoplastic effects in cervical cancer cells by causing apoptosis, inhibiting migration, and modulation of genes related to apoptosis and migration. The lower concentrations of cisplatin, chrysin, and luteolin were taken for testing combinations. The highest concentration combinations of having C3, CH3, and L3 were not used for molecular level studies as our aim was to depict the best effect taking lower concentrations of the used agents. However, a combination of topotecan with chrysin and luteolin depicted antagonism. The literature has also depicted synergistic as well as antagonistic effects between various chemopreventive and chemotherapeutic agents. Hence, it is also important to investigate the antagonist effects of various combinations because some drug and even dietary combinations may have a negative effect on human health. The investigation of their inflection of various molecular targets will further add to the mechanistic knowledge that can be applied to pinpoint the most effective combination therapy.

Further, this study clearly demonstrated the significant potential of combination studies combining natural medicines with chemotherapeutic drugs. Such beneficial combinations will make it easier to design a multifaceted therapeutic strategy, which could aid in overcoming the drawbacks of chemotherapy. However, the limitation of this study is that it depicts effects in vitro only; additional, in vivo investigation of these combinations is necessary and will permit a thorough assessment of their outcomes.
